# Thermal post-treatment alters nutrient release from a controlled-release fertilizer coated with a waterborne polymer

**DOI:** 10.1038/srep13820

**Published:** 2015-09-08

**Authors:** Zijun Zhou, Changwen Du, Ting Li, Yazhen Shen, Jianmin Zhou

**Affiliations:** 1 State Key Laboratory of Soil and Sustainable Agriculture, Institute of Soil Science, Chinese Academy of Sciences, Nanjing 210008, China; 2 College of Resources, Sichuan Agricultural University, Chengdu 611130, China; 3 University of Chinese Academy of Sciences, Beijing 100049, China

## Abstract

Controlled-release fertilizers (CRF) use a controlled-release technology to enhance the nutrient use efficiency of crops. Many factors affect the release of nutrients from the waterborne polymer-coated CRF, but the effects of thermal post-treatments remain unclear. In this study, a waterborne polyacrylate-coated CRF was post-treated at different temperatures (30 °C, 60 °C, and 80 °C) and durations (2, 4, 8, 12, and 24 h) after being developed in the Wurster fluidized bed. To characterize the polyacrylate membrane, and hence to analyze the mechanism of nutrient release, Fourier transform mid-infrared spectroscopy, scanning electron microscopy, and atomic force microscopy were employed. The nutrient-release model of CRF post-treated at 30 °C was the inverse “L” curve, but an increased duration of the post-treatment had no effect. The nutrient-release model was “S” curve and nutrient-release period was enhanced at higher post-treatment temperatures, and increased post-treatment duration lengthened slowed nutrient release due to a more compact membrane and a smoother membrane surface as well as a promoted crosslinking action. CRF equipped with specified nutrient-release behaviors can be achieved by optimizing the thermal post-treatment parameters, which can contribute to the development and application of waterborne polymer-coated CRF and controlled-release technologies.

Controlled-release technology has become popular in recent decades, especially in the pharmaceutical industry and agriculture^1^,^2^,^3^. In agriculture, the technology is mainly used for fertilizers and pesticides^4^. Currently, food security is a worldwide issue as a result of exponential population growth and diminishing areas of arable land^5^,^6^. To maintain or enhance crop yields per hectare, excessive fertilizer is often applied, but this has seriously impacted the environment, human health, energy and resource conservation targets, and it constrains sustainable agricultural development^7^,^8^.

Controlled-released fertilizers (CRF) enhance nutrient use efficiency, reduce labor costs associated with fertilization, minimize negative toxic effects of excessive fertilization, and aim to supply available nutrients to coincide with plant demand for a long controlled-release period^9^,^10^. There are many factors in controlled-release technology that affect the mechanism of nutrient release. The coating materials and the coating process are two key factors that contribute to either a “sudden release” or a “diffusion release” of nutrients from the CRF^11^,^12^.

Sulfur and polymer are two kinds of coating materials for CRF on a commercial scale, and they have been used by many organizations, such as Tennessee Valley Authority in USA, Agrium Inc. in Canada, Haifa Chemicals Co. Ltd. in Israel, and Shandong Kingenta Ecological Engineering Co. Ltd. in China^13^,^14^,^15^,^16^. However, compared with sulfur coated CRF, polymer-coated CRF have recently become more commercially successful due to their improved controlled-release rate, release time, and release pattern^17^,^18^. Furthermore, in comparison with the traditional organic coating, waterborne polymer-coated materials are economical, of high quality, safe to use, and environmentally friendly^2^,^19^,^20^.

The Wurster fluidized bed is preferred coating machine over the pan, rotary drum, and conventional fluidized beds because it produces high-quality, rapidly drying CRF of uniform thickness^12^. There are more than 20 operational parameters involved in the Wurster fluidized bed coating process^21^, and some of the key parameters in the spray coating process have been analyzed (e.g., spray rate of the polymer latex, fluidization air velocity, atomizing gas flow rate, and gas temperature) and found to significantly affect coating morphology and quality, which in turn affect the behavior of the CRF^12^,^22^.

Optimization of the thermal post-treatment parameters after the polymer coating process is still necessary. However, to the best of our knowledge, there is little information on the thermal post-treatment parameters for waterborne polymer-coated CRF, except for Zhao *et al.*^19^ who found that higher temperatures strengthened the hydrophobicity of the CRF model membrane which was developed statically in an oven. In our current study, the objective was to examine the effects of thermal post-treated parameters on the nutrient-release behavior of a waterborne polyacrylate-coated CRF. The CRF was produced in a Wurster fluidized bed and then post-treated in a convection oven. Nutrient release from the CRF was detected in distilled water, and the membrane changes were observed using Fourier transform mid-infrared spectroscopy (FT-IR), scanning electron microscopy (SEM), and electron digital caliper and atomic force microscopy (AFM).

## Results

### Nutrient release from the polymer-coated CRF

**Figure 1:**
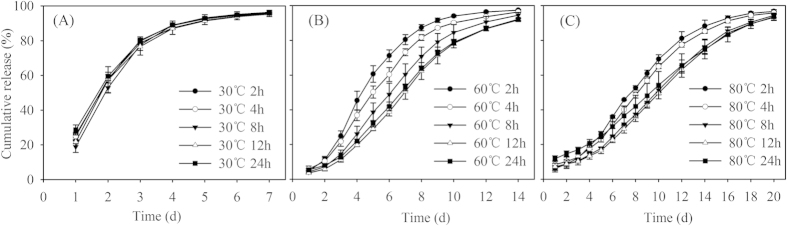
Cumulative nutrient-release profiles from the controlled-release urea post-treated at 30 °C **(A)**, 60 °C **(B)**, and 80 °C **(C)** for 2, 4, 8, 12, and 24 h, factorially, over the incubation time at 25 °C in static distilled water. Bars indicate standard error of the mean (n = 3). Treatment details are given in Methods.

The profiles of nutrient release into distilled water from the post-treated CRF at 30 °C, 60 °C, and 80 °C for 2, 4, 8, 12, and 24 h, factorially, are shown in Fig. 1. The fastest nutrient release occurred for the 30 °C post-treatment with an 80% cumulative release at 3 d, and increased post-treatment time did not significantly change the nutrient release. Higher post-treatment temperatures (60 °C and 80 °C) slowed the controlled release, and changed the release model from the inverse “L” curve at 30 °C to the “S” curve at both 60 °C and 80 °C. The cumulative release reached 80% at about 7, 8, 9, and 10 d for the 60 °C post-treatment applied for 2, 4, 8, and 12 h, respectively; the 24 h post-treatment did not extend the duration of release. The cumulative release reached 80% at 12 d for the 80 °C post-treatments applied for 2 and 4 h, and at about 15 d for post-treatments applied for 8, 12, and 24 h.

### Membrane characteristics of the polymer-coated CRF

**Figure 2:**
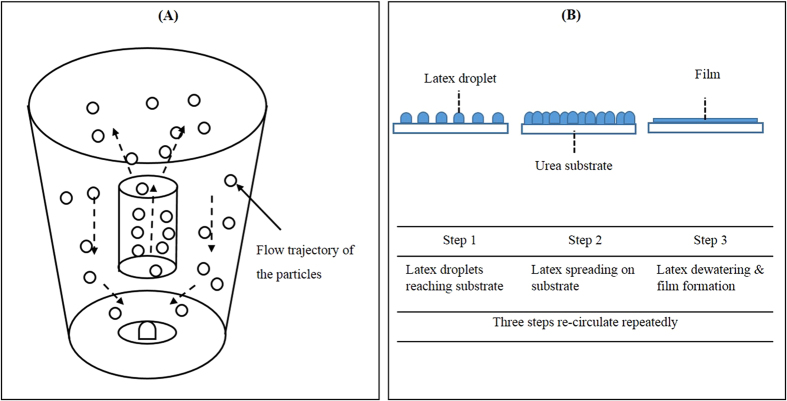
Schematic of the spray coating process in a Wurster fluidized bed^12^ **(A)** and film formation in the spray coating process^22^ **(B)**.

During the Wurster fluidized bed procedure, waterborne polymer emulsion was sprayed onto the surface of the round urea granules to form a coating that was immediately dried. Repeated spraying of the emulsion and then drying built a membrane coating around the granules^12^,^22^ (Fig. 2).

**Figure 3:**
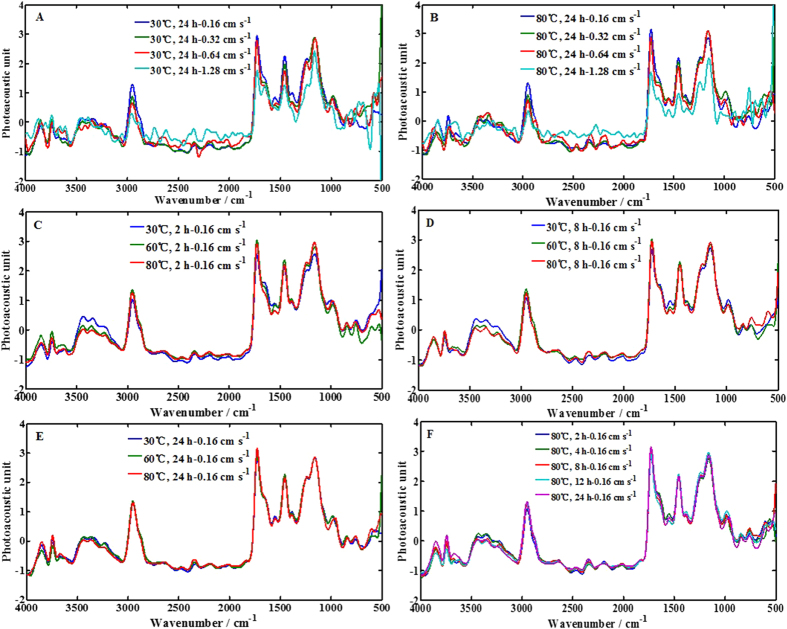
FT-IR spectra of the coating membranes from the waterborne polyacrylate-coated CRF under different post-treated temperatures and times. (**A**,**B**), spectra of membranes at four mirror velocities post-treated under 30 °C (**A**) and 80 °C (**B**) for 24  h each; (**C**–**F**), spectra of CRF membranes at 0.16 cm s^−1^ moving mirror velocity that were post-treated at three temperatures (30 °C, 60 °C, and 80 °C) for 2 h (**C**), 8 h (**D**), and 24 h (**E**), and at 80 °C for five durations (2, 4, 8, 12, and 24 h) (**F**).

The FT-IR was used to detect the composition of the coating membrane. The FT-IR spectra of the coating membranes had several similar functional groups: O–H and N–H stretching vibration (3250–3550 cm^−1^), C–H stretching vibration (~2850 cm^−1^), C = O stretching vibration (~1730 cm^−1^), C–H bend vibration (~1450 cm^−1^), and C–O stretching vibration (~1160 cm^−1^) (Fig. 3). However, there were some differences among the spectra. Calculations were made for the profiling depth of the membranes:


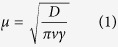


where *μ* is the profiling depth (μm), *D* is the thermal diffusivity of sample (m^2^ s^−1^), *v* is the moving mirror velocity (cm s^−1^), and γ is the wavenumber (cm^−1^)^23^. The profiling depths (μm) were calculated at four different moving-mirror velocities (0.16, 0.32, 0.64, and 1.28 cm s^−1^), whereby 0.16 cm s^−1^ was the deepest layer, and 1.28 cm s^−1^ was the outermost layer. The coating membrane was heterogeneous (Fig. 3A,B). Membrane hydrophobicity was a key factor affecting the nutrient-release pattern from the CRF, and it was generally acknowledged that waterborne polymer coated CRF with higher membrane hydrophobicity had a longer controlled period of nutrient-release^19^,^24^. The hydrophilic groups, such as the –OH and –NH, and the hydrogen bond acceptors, such as the carbonyl of ester, facilitated moisture uptake. The ratio of intensity of hydrophobic group (–CH) to the intensity of hydrophilic group (e.g., –NH, –OH, and –C = O) could be used to revealed the hydrophobicity of the membrane^19^. Therefore, spectra at 0.16 cm s^−1^ were further used for their high signal-to-noise ratio and high hydrophobicity. Figure 3C–E show the changes in the membrane spectra during the post-treatments at 30 °C, 60 °C, and 80 °C, respectively. From the spectra, the membranes post-treated at 30 °C showed the largest intensity of hydrophilic group (e.g., –NH, –OH, and –C = O) with the least intensity of hydrophobic group (–CH) in three post-treated temperatures, which meant membranes post-treated at 30 °C was the least hydrophobic in three temperatures for the 2 h and 8 h of post-treatment duration; and hydrophobicity of membranes post-treated at 80 °C was slightly higher than that at 60 °C. The hydrophobicity of the membranes exposed to different post-treatment temperatures became more similar as the post-treatment duration increased, which was attributed to the both the time necessary to complete the crosslinking action (about 1 d at room temperature)^25^, and the high temperature to accelerate the action. Figure 3F shows that lower intensity of hydrophilic group (e.g., –NH, –OH, and –C = O) and higher intensity of hydrophobic group (–CH) resulted from increased thermal post-treated duration at post-treated temperature of 80 °C, which means the crosslinking action needed time for completion.

**Figure 4:**
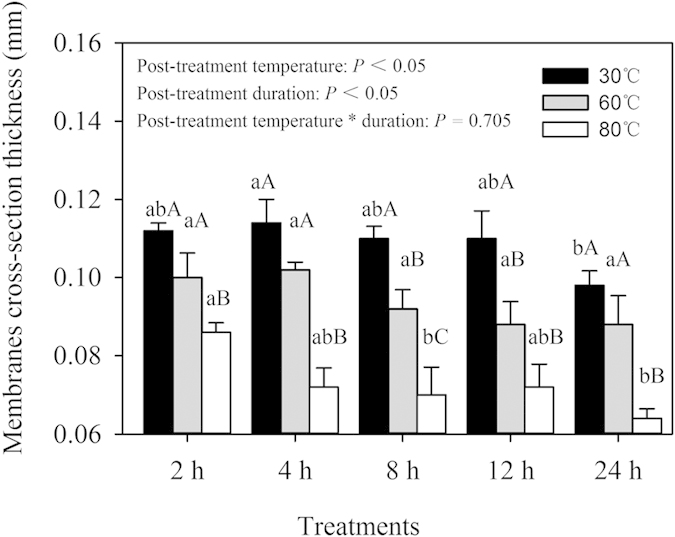
Cross-section thickness of the polymer membrane on the CRF post-treated at 30 °C, 60 °C, and 80 °C for 2, 4, 8, 12, and 24 h, factorially. Bars indicate standard error of the mean (n = 5). Treatment details are given in Methods. Values with different lowercase letters are different at a significance level of *P* < 0.05 for each post-treatment temperature. Values with different uppercase letters are different at a significance level of *P* < 0.05 for each post-treatment duration. The interaction between post-treatment temperature and duration had no significant effect on membrane cross-section thickness (*P* = 0.705).

**Figure 5:**
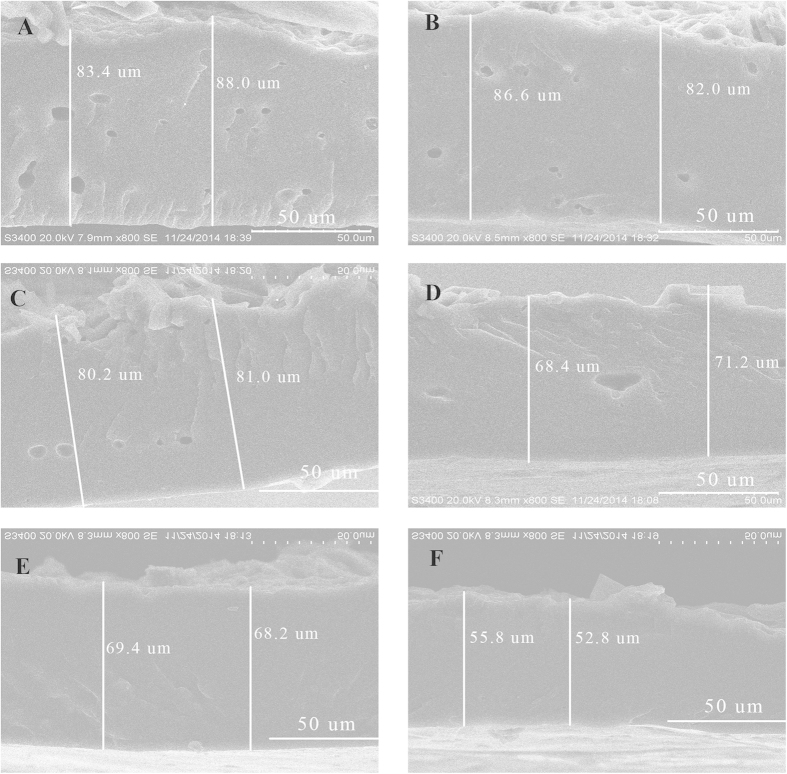
Cross-section images of coating membranes from waterborne polyacrylate-coated CRF under different post-treatment temperatures and durations using a scanning electron microscopy (SEM). (**A**–**F**), membranes of post-treatments at 30 °C 2 h (**A**), 30 °C 8 h (**B**), 60 °C 2 h (**C**), 60 °C 8 h (**D**), 80 °C 2 h (**E**), and 80 °C 8 h (**F**), respectively.

An electron digital caliper and SEM were employed to reveal the cross-section structure of the coating membrane. Figure 4 shows that using an electron digital caliper, the cross-section thickness of the coating membrane decreased as the post-treatment temperature and duration increased. The CRF post-treatment at 30 °C for 2 h was the thickest (~0.11 mm), whereas that treated at 80 °C for 24 h was the thinnest (~0.07 mm). Using SEM, we examined coating membranes post-treated at 30 °C, 60 °C, and 80 °C for 2 h and 8 h each, to demonstrate cross-section morphological structures (Fig. 5). The membrane cross-section thickness of CRF post-treated at 30 °C was the thickest (~0.085 mm), whereas that at 80 °C for 8 h was the thinnest (~0.055 mm). The trends for the cross-section thicknesses in response to increased post-treatment temperature and duration were similar between the two measurement methods, although the electron digital caliper indicated thicker membranes than those measured using SEM. This might be attributed to inconsistent whole membrane thickness, or fertilizer dust adhering to the membrane. Furthermore, cross-sections of membranes post-treated at 30 °C for 2 h showed the largest number of pores, whereas no observable pores were observed in cross-sections of membranes post-treated at 80 °C. Therefore, the pore size and pore density of the post-treated membranes significantly decreased with an increase in post-treatment temperature and duration, and increased temperature significantly compacted the coating membrane.

**Figure 6:**
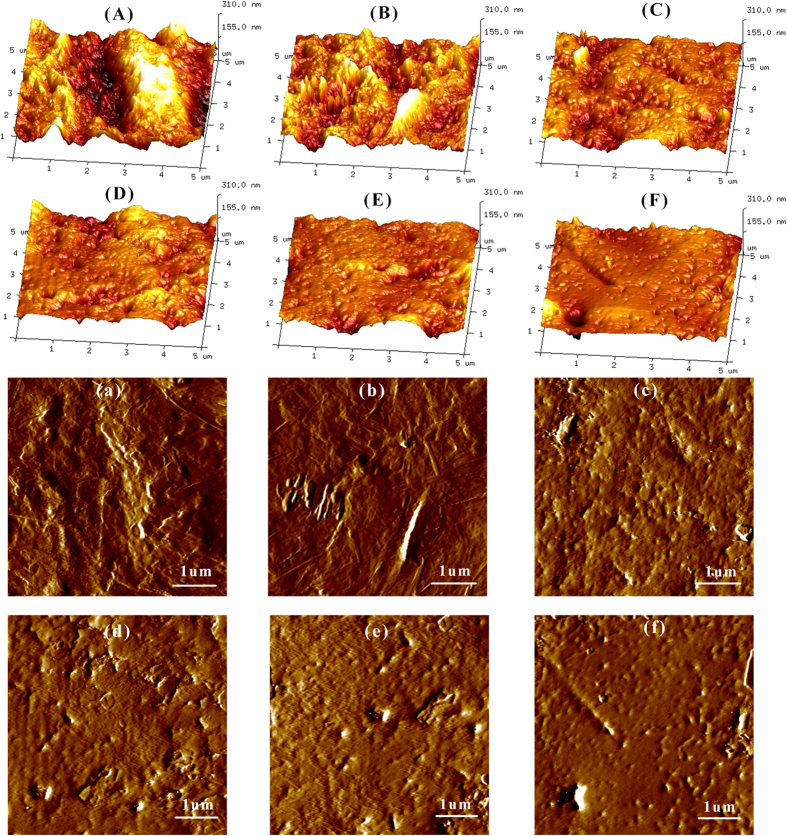
Surface morphological structures of membranes from CRF post-treated at 30°C, 60°C, and 80°C for 2 h and 8 h, factorially. (**A**–**F**): height images of membranes post-treated at 30 °C for 2 h (**A**), 30 °C for 8 h (**B**), 60°C for 2 h (**C**), 60 °C for 8 h (**D**), 80 °C for 2 h (**E**), and 80 °C for 8 h (**F**); a–f: phase images of membranes post-treated at 30 °C for 2 h (**a**), 30 °C for 8 h (**b**), 60°C for 2 h (**c**), 60 °C for 8 h (**d**), 80 °C for 2 h (**e**), and 80 °C for 8 h (**f**).

AFM measurements were used to study the surface morphology of membranes from a CRF post-treated at 30 °C, 60 °C, and 80 °C for 2 h and 8 h, respectively. Figure 6 shows the three dimensional height images (A–F) and phase images (a–f) from AFM. It can be clearly observed from the three dimensional height images as well as the phase images that the membranes post-treated at 60 °C and 80 °C were smoother than those at 30 °C. Table 1 represents the average roughness (Ra), root mean square roughness (Rq), and maximum roughness (Rmax), which were calculated using AFM software (NanoScope Analysis). There was a similar decreasing trend in membrane Ra, Rq, and Rmax from a CRF post-treated at 30 °C for 2 h and 8 h, 60 °C for 2 h and 8 h, and 80 °C for 2 h and 8 h. Membranes post-treated at 30 °C were the roughest, whereas membrane roughness was no different between 60 °C and 80 °C. Higher temperatures may contribute to polymer remodeling, and lead to more compact membranes with smoother surfaces.

## Discussion

The type of coating material and the coating technology are both responsible for the mechanism of nutrient release from controlled-release fertilizers^11^,^12^. In this study, we concluded that thermal post-treatment parameters were closely related to the nutrient-release behavior of the waterborne polymer-coated CRF. The temperature of the fluidizing gas must be high enough to evaporate the solvent, and to prevent aggregation of the coated particles, and it also should be below an upper-temperature (e.g., polymer-softening temperature) to avoid massive aggregation^13^. Lan *et al.*^22^ found that the gas temperature significantly affected the film surface morphology and that sprayed films prepared at 35 °C were smooth, and enhanced gas temperature led to more porous structures in the films, which resulted in increased film permeability coefficients when the gas temperature was higher than 40 °C. Thompson and Kelch^26^ reported that process temperatures that were 20 °C–30 °C higher than the latex glass temperature was suitable for the formation of a dense and uniform film. Therefore, in our study, a gas temperature of about 35 °C was adopted to develop the CRF in the Wurster fluidized bed.

Kong *et al.*^27^ and Pacheco *et al.*^28^ found that membrane structural factors (thickness, pore size, surface area, and roughness) and chemical properties (hydrophilicity or hydrophobicity) affected the water flux. The hydrophobicity mainly depended on the following factors: hydrophilic groups present in the polymer, Vander Waals’ interaction in terms of hydrogen bonding, average molecular interchain structure, and hydrophobicity of the alkyl chain length^29^. These are possible reasons for the different release behaviors from the CRF exposed to various post-treatments.

For the CRF post-treatment at 30 °C, abundant pores in the membrane cross-section contributed to the water molecules quickly moving into the CRF coating and to the easy release of small urea molecules from the pores, which resulted in a high rate of nutrient release. High surface roughness of the membrane also led to low hydrophobicity^30^ and increased water contact with the higher surface area of the membrane^31^, which also accelerated nutrient release. Post-treatment duration did not affect the controlled-release behavior, mainly because pore size and membrane cross-section thickness did not change significantly at the relative low temperature; although aziridine ring groups of the cross-linker can react with –COOH in waterborne polyacrylate emulsion at room temperature (e.g., 30 °C).

For CRF post-treatment at 60 °C and 80 °C, the pore density in the membrane cross-section, the surface roughness of the membrane were all reduced significantly compared with CRF post-treated at 30 °C. This contributed to changing the nutrient controlled-release model from inverse “L” curve to “S” curve and prolonging the nutrient-release period. With an increase in post-treatment duration (from 2 h to 8 h) at 60 °C, pore density and the cross-section thickness of the membrane reduced significantly, which led to a compact membrane, which slowed the nutrient release.

Nutrient-release from the CRF post-treated at both 60 °C and 80 °C slowed with an increase in the post-treatment duration, but there was no additional slowing of nutrient release at 60 °C after 12 h or at 80 °C after 8 h of the post-treatment, which may be attributed to the completion of crosslinking action and polymer remodeling. Compared with CRF post-treated at 30 °C and 60 °C, there were several reasons for the slower nutrient release for the CRF post-treated at 80 °C. Firstly, no pores occurred in the membrane cross-section and the membrane cross-section was the thinnest for this treatment, which meant that the membrane from the CRF post-treated at 80 °C was the most compact. Secondly, the higher temperature promoted crosslinking action and consequently strengthened the membrane hydrophobicity^19^, which decreased the ability of water molecules to be absorbed. In addition, high temperature could enhance the mechanical strength of the membrane^32^, which may reduce the nutrient-release surface area of the swollen CRF.

The different nutrient-release models for CRF under varied post-treated parameters indicated that a higher temperature led to a more compact membrane with less pores in the cross-section and smoother membrane surface, and then might reduce the film diffusivity. CRF equipped with specified nutrient-release behaviors could be achieved by specified adjustments of post-treatment parameters, which could contribute to the development and application of waterborne polymer-coated CRFs and controlled-release technologies.

## Methods

### Materials

To prepare the CRF, commercial grade urea granules (46.4% nitrogen) were purchased from Luxi Chemical Co. Ltd. (Liaocheng, China), and waterborne polyacrylate emulsion (50% dry matter content), provided by Doctor Hydrophilic Chemicals Co. Ltd. (Yizheng, China), was used as a coating material. The emulsion was polymerized from butyl acrylate, methyl methacrylate, and methyl acrylic acid. The glass transition temperature of the polymer is around 8 °C^19^.

### Preparation of the polymer-coated CRF

Urea granules (36 kg) were loaded into a pilot scale Wurster fluidized bed (LDP-5, Jiafa Mechanic Co. Ltd., Changzhou, China). The bed temperature was set at 35 ± 5 °C and preheated for 10 min. Waterborne polyacrylate emulsion (8 kg) was diluted with 8 kg water and then mixed with 80 g of the cross-linker aziridine, which was sprayed through a nozzle using a peristaltic pump at the speed of 0.20 L min^−1^ at an atomizing pressure of 0.4 MPa. A 2 kg batch of coated CRF was stored at 4 °C in a desiccator containing allochroic silica gel^19^,^22^.

### Characterization of nutrient release from the CRF

The CRF was subdivided into 15 samples, which were post-treated at three temperatures (30 °C, 60 °C, and 80 °C) for five durations (2, 4, 8, 12, and 24 h), factorially. A 10 g sample of CRF granules was placed in a sealed glass bottle with 200 mL of distilled water and then incubated at 25 °C. The nutrient release was measured using the paradimethylaminobenzaldehyde colorimetric method every 24 h for the first 10 days and then every 48 h until the end of the experiment. The distilled water in the glass bottle was refreshed after measuring the released nutrients. On the last day of nutrient release, the coated fertilizers were ground to determine the residual nutrients. The nutrient-release profile was estimated as the cumulative release percentage versus the incubation time.

### Characterization of the CRF membrane

The post-treated CRF granules were cut into two pieces with a sharp blade, and the coating membranes were removed slightly from the CRF granules with tweezers.

To determine the composition of the coating membranes we used an FT-IR spectrometer (Nicolet 6700, MA, USA) with a photoacoustic accessory (MTEC model 300, IA, USA). The scans were conducted in the mid-IR region (500–4000 cm^−1^) with a resolution of 4 cm^−1^ and four mirror velocities of 0.16, 0.32, 0.64, and 1.28 cm s^−1^, and 32 successive scans were recorded. A glassy carbon reference material was used as the surface absorber to calibrate the system^33^.

An AFM (Dimension Edge, Bruker, Germany) was used to study the surface topography of the membranes. The membranes were fixed onto the mica plate and then positioned on top of the scanner tube. The AFM laser beam was focused onto the preselected spot of the surface prior to engaging the cantilever. AFM images were collected in tapping mode with silicone tip cantilevers^34^.

The cross-section thickness of the coating membranes were measured using an electron digital caliper (Chengliang Tools Group Co. Ltd, Chengdu, China). The cross-section morphology of the coating membranes were examined by SEM (S-3400, Hitachi, Japan) with an accelerating voltage of 20 kV and 95 μA. The coating membranes were fractured in liquid nitrogen and cross-sections of the samples were gold sputtered for 2 min before observation.

### Data analyses

All spectral data were processed using Matlab 2009b. The AFM images were analyzed using the NanoScope software (Version 5.12b48). A two-way analysis of variance (ANOVA) was performed to test the primary and interactive effects of the post-treatment temperature and duration on both membrane cross-section thickness (Fig. 4) and membrane roughness (Table 1) using SPSS 16.0. The assumption of homogeneity of variance was tested with Levene’s test at a = 0.05. When statistically significant differences existed according to ANOVA (P < 0.05), treatment means were compared using Duncan test at a = 0.05.

## Additional Information

**How to cite this article**: Zhou, Z. *et al.* Thermal post-treatment alters nutrient release from a controlled-release fertilizer coated with a waterborne polymer. *Sci. Rep.* **5**, 13820; doi: 10.1038/srep13820 (2015).

## Figures and Tables

**Table 1:** Surface roughness (Mean ± Standard Error of Mean, n = 3) of the membranes from the CRF post-treated at 30 °C, 60 °C, and 80 °C for 2 and 8 h, factorially, by atomic force microscopy (AFM).

**Post-treatment temperatures**	**Ra (nm)**		**Rq (nm)**		**Rmax (nm)**	
	**2** **h**	**8** **h**	**2** **h**	**8** **h**	**2** **h**	**8** **h**
30 °C	30.1 ± 3.7A	25.8 ± 3.7A	39.6 ± 3.5A	33.5 ± 4.9A	281.3 ± 10.7A	284.0 ± 44.0A
60 °C	12.9 ± 1.4B	12.5 ± 1.5B	17.2 ± 1.5B	16.9 ± 1.7B	152.7 ± 13.3B	159.7 ± 7.1B
80 °C	14.0 ± 1.4B	12.1 ± 2.3B	16.9 ± 2.7B	16.0 ± 3.2B	161.2 ± 44.3B	160.6 ± 35.6B

Ra: average roughness; Rq: root mean square roughness; Rmax: maximum roughness. Values with different letters are different at a significance level of *P* < 0.05 for each post-treatment duration. The different post-treatment durations had no significant effect on membrane Ra (*P* = 0.219), Rq (*P* = 0.270) and Rmax (*P* = 0.883), respectively. The interaction between post-treatment temperature and duration had no significant effect on membrane Ra (*P* = 0.649), Rq (*P* = 0.489) and Rmax (*P* = 0.988), respectively.
